# Gaming Device Usage Patterns Predict Internet Gaming Disorder: Comparison across Different Gaming Device Usage Patterns

**DOI:** 10.3390/ijerph14121512

**Published:** 2017-12-05

**Authors:** Soo-Hyun Paik, Hyun Cho, Ji-Won Chun, Jo-Eun Jeong, Dai-Jin Kim

**Affiliations:** Department of Psychiatry, Seoul St. Mary’s Hospital, The Catholic University of Korea, Seoul 06591, Korea; suehime@gmail.com (S.-H.P.); sonap1@hanmail.net (H.C.); cuneus@naver.com (J.-W.C.); goodi11@hanmail.net (J.-E.J.)

**Keywords:** Internet gaming disorder, game device usage pattern, smartphone, comorbidity

## Abstract

Gaming behaviors have been significantly influenced by smartphones. This study was designed to explore gaming behaviors and clinical characteristics across different gaming device usage patterns and the role of the patterns on Internet gaming disorder (IGD). Responders of an online survey regarding smartphone and online game usage were classified by different gaming device usage patterns: (1) individuals who played only computer games; (2) individuals who played computer games more than smartphone games; (3) individuals who played computer and smartphone games evenly; (4) individuals who played smartphone games more than computer games; (5) individuals who played only smartphone games. Data on demographics, gaming-related behaviors, and scales for Internet and smartphone addiction, depression, anxiety disorder, and substance use were collected. Combined users, especially those who played computer and smartphone games evenly, had higher prevalence of IGD, depression, anxiety disorder, and substance use disorder. These subjects were more prone to develop IGD than reference group (computer only gamers) (B = 0.457, odds ratio = 1.579). Smartphone only gamers had the lowest prevalence of IGD, spent the least time and money on gaming, and showed lowest scores of Internet and smartphone addiction. Our findings suggest that gaming device usage patterns may be associated with the occurrence, course, and prognosis of IGD.

## 1. Introduction

Playing online games is one of the most popular recreational activities. According to a Korean national survey conducted in 2016, 67.9% of the general population aged from 10 to 65 years old played online games [1]. Though gaming is a pleasurable and stimulatory activity, the dark side of excessive gaming is evident as well. Excessive use and loss of control over gaming has brought about various mental health and social concerns [2]. As numerous psychological and neurobiological correlates of excessive Internet gaming have been elucidated, such as impulsivity, reward sensitivity, and altered brain structure and function [3,4], the revised version of the Diagnostic and Statistical Manual of Mental Disorder, fifth edition (DSM-5) has listed the phenomenon of Internet gaming disorder (IGD) as a condition for further research [5]. 

To date, studies on IGD have mainly focused on personal computer (PC) games, especially massive multiplayer online role-playing games (MMORPGs) [3], and consistently reported that IGD was highly prevalent among male adolescents [6]. However, the spread of smartphones has explosively increased the number of gamers in female and in all age groups and subsequently changed the demographics and characteristics of online gamers. According to a Korean national survey conducted in 2015, among Korean smartphone users, 86.5% of the forties, 85.4% of the fifties, and 88.8% of females reported they had experiences of playing online games [7]. In addition, recent advances in smartphone game platforms have increased accessibility to various game genres including real-time strategy, MMORPG, or shooting games. Accordingly, the number of gamers who played with both devices has rapidly grown as well: 56.9% of the responders played smartphone games only while 20% played PC games only, and the remaining played both PC and smartphone games [7]. 

Given that each device has unique interface features and characteristics, gaming device usage patterns, such as single or combined use or time dedicated to each device, may play an important role on gaming behaviors and clinical characteristics and the occurrence of IGD. However, only a few studies have investigated the role of gaming device usage patterns on gaming-related attitudes or comorbid psychopathology. This study was designed to explore gaming behaviors and clinical characteristics across different gaming device usage patterns and their role on IGD. 

## 2. Materials and Methods 

### 2.1. Participants and Procedures

Data was collected from a large online survey conducted between April and September 2016 on online gaming and smartphone usage behaviors. Participants aged from 14 to 39 years were recruited from a pool of panelists registered for online panels at Panel Marketing Interactive (PMI), a research company that provides survey-related technology and data collection. The participants were given tokens that could be used as cybermoney as an incentive for their participation. In total, 9474 people were contacted and ultimately 7200 people (76% of those contacted) participated in our online survey. From the total 7200 responders, adults aged from 20 to 39 who both played online games and owned smartphones were included in this study (*n* = 3470). Since adolescents are considered to be more vulnerable to addictive disorders due to high novelty seeking and risk-taking temperament during adolescent period and immature cortical growth, which plays a critical role in cognitive control [8,9], we decided that the adult sample should be analyzed separately from adolescents. Though about 80% of smartphone users aged over 40 years had experiences of playing online games, the number of PC-based online gamers in this age are much lower than younger adults [1]. Thus, we set the upper age limit as 40 years old. We excluded responders who used game consoles (*n* = 412, 11.8% of total responders) for two reasons: (1) most of them also played PC and smartphone games (397, 96.3%), and (2) the aim of this study was to investigate the gaming characteristics among PC and smartphone gamers. Finally, 3058 subjects were selected (1548 males and 1510 females). The mean age was 26.95 years (standard deviation (SD) = 5.859 years). 

All study procedures were performed in accordance with the guidelines of the Declaration of Helsinki. The Institutional Review Boards of Seoul St. Mary’s Hospital approved the study protocol (KC15EISI0103). All subjects were informed about the study and all provided informed consent.

### 2.2. Measures

#### 2.2.1. Demographic and Gaming Characteristics

Demographic information on education levels and occupation status was asked. Education levels were classified into two categories: (1) up to 12 years (up to high school graduates), and (2) more than 13 years (currently in university/college or higher education). Occupational status was classified into three categories: (1) current students; (2) individuals currently with full-time jobs, and (3) individuals currently without full-time jobs. 

All participants were asked to answer dichotomously (1: Yes, 2: No) to nine diagnostic criteria questions for IGD according to the DSM-5. Participants who answered “yes” to five or more criteria for questions pertaining to the previous 12 months were defined as the IGD group in line with previous studies [10,11,12], and those with four or less affirmative answers were defined as the non-IGD group. Gaming device usage patterns were determined by reports from the responders on the time proportion dedicated to either PC or smartphone (SM) and were classified into five groups: (1) PC only group: individuals who played only PC games, 100% dedicated to PC games (*n* = 720); (2) PC > SM group: individuals who played more PC games than SM games, 60% to 99% dedicated to PC games and 1% to 40% to SM (*n* = 580); (3) PC = SM group: individuals who played PC and SM games evenly, 41% to 59% dedicated to both PC and SM games (*n* = 326); (4) PC < SM group: individuals who played SM games more than PC games, 60% to 99% dedicated to SM games and 1% to 40% to PC (*n* = 735), and (5) SM only group: individuals who played only SM games, 100% to SM games (*n* = 697). Additionally, time (minutes) and money (by Korean currency, KRW, per month) spent on gaming, whether they owned game community memberships, and whether they have any experience of attending offline meetings were asked. The most preferred game was asked and classified into five genres according to the White Paper on Korean Games [13]: (1) simulation and real-time strategy (i.e., League of Legends, StarCraft, and the Sims); (2) role-playing game (RPG) (i.e., World of Warcraft and Lineage); (3) sports and racing (i.e., FIFA, Winning Eleven, Need for Speed, and Tales Runner); (4) shooting and action (i.e., Sudden Attack, Counter Strike, and Virtual Fighter) and (5) puzzle, arcade, and board games (i.e., Candy Crush Saga, Monopoly, and rhythm games). The main motive for gaming was selected from the following categories: (1) for fun; (2) for killing time; (3) for relieving stress; (4) for need (i.e., to maintain interpersonal relationship), and (5) for sense of achievement. 

#### 2.2.2. Clinical Characteristics

Participants were asked to answer the following questionnaires in order to determine clinical characteristics: Young’s Internet Addiction Test (YIAT), Smartphone Addiction Scale-Short Version (SAS-SV), Brief Self-Control Scale (BSCS), Patient Health Questionnaire-9 (PHQ-9), Generalized Anxiety Disorder-7 (GAD-7), Alcohol Use Disorder Identification Test (AUDIT), and Fagerstrom Test for Nicotine Dependence (FTND).

YIAT, a 20-item scale which is rated by a five-point Likert scale (1: Not at all, 5: Almost always) [14], was used to assess the severity of Internet addiction [15]. In South Korea, YIAT had acceptable internal consistency and reliability (Cronbach’s alpha = 0.921) [16] and Cronbach’s alpha for YIAT was 0.956 in this sample. 

SAS-SV, a 10-item scale which is rated by a six-point Likert scale (1: Strongly disagree, 6: Strongly agree), was used to assess the severity of smartphone addiction [17]. SAS-SV has excellent concurrent validity and highly correlated with the original version (Cronbach’s alpha = 0.958, *p* < 0.001). SAS-SV has been used in adult sample to assess the degree of smartphone addiction across various countries; a higher score indicated a higher degree of smartphone addiction [18,19]. Cronbach’s alpha was 0.773 in this study.

BSCS, a 13-item questionnaire with a five-point Likert scale (1: Strongly disagree, 5: Strongly agree), was used to measure self-control ability. BSCS measures the ability to override or change one’s inner response as well as to interrupt undesired behavioral tendencies and refrain from acting on them, with higher scores indicating lower self-control ability. BSCS had good internal consistency (Cronbach’s alpha = 0.85) in the original study [20]. Cronbach’s alpha was 0.757 in this study.

PHQ-9 is a nine-item depression rating scale which corresponds to the major depressive episode criteria of the DSM-IV with a four-point Likert scale (0: Not at all, 3: Almost every day) [19]. We defined individuals with scores of 10 or more as having depression according to Manea and colleagues [21]. The Korean version of PHQ-9 has proven to have excellent validity and reliability in primary care patients for detecting major depressive disorder (Cronbach’s alpha = 0.852) [22]. Cronbach’s alpha was 0.893 in this study.

GAD-7, a seven-item scale with a four-point Likert scale (0: Not at all, 3: Nearly every day), was used to screen generalized anxiety disorder (GAD) [23]. GAD-7 is particularly useful in assessing symptom severity. We defined individuals with scores of 10 or greater as having GAD according to Plummer and colleagues [24]. GAD-7 had excellent internal consistency (Cronbach’s alpha = 0.92) [23]. Cronbach’s alpha was 0.908 in this study.

AUDIT, developed by the World Health Organization, is a 10-item scale to identify alcohol-related problems [25]. Total scores range from 0 to 40 and the optimal cut-off point to identify at-risk alcohol users is 10 for males and 6 for females in South Korea [26]. In this study, alcohol use disorder (AUD) was defined for males as a score of 10 or more and for females as a score of 6 or more. AUDIT had good internal consistency (Cronbach’s alpha = 0.80–0.93) and Cronbach’s alpha was 0.859 in this study.

FTND is a six-item scale which is most widely used to measure nicotine dependence [27]. Total scores range from 0 to 10 and individuals with scores of four or more were determined to have nicotine dependence [28]. The Korean version of FTND was standardized and Cronbach’s alpha was 0.6913, which was similar in this study, 0.636.

### 2.3. Statistical Analysis

The quantitative variables are presented as means ± SD and the qualitative data are presented as absolute numbers (N) and percentages (%). To compare differences between IGD and non-IGD groups, and across different gaming device usage pattern groups, non-parametric Mann-Whitney U tests and Kruskal-Wallis H tests were used, respectively. Additional post-hoc tests were done by the Mann-Whitney U tests. To examine the predictive values of each variable for IGD, binary logistic regression analysis was performed and the results are shown by odds ratios (ORs) and 95% confidential intervals (CIs). All statistical works were performed by using SPSS version 24.0 (SPSS Inc., Chicago, IL, USA).

## 3. Results

### 3.1. Comparison between the IGD and Control Groups

Table 1 shows differences in gaming behaviors and clinical characteristics between IGD and non-IGD subjects. From the total 3058 participants, 396 (12.9%) were classified as having IGD. IGD subjects had higher mean age, were more likely to be males, had lower prevalence of participants with higher education or who currently had full-time jobs, spent more time and money on gaming, and had more game community membership and experience of offline meeting attendance than non-IGD subjects. IGD was more prevalent in combined user groups (PC > SM, PC = SM, and PC < SM groups) than in the single user groups (PC and SM only groups) (66.4% vs. 51.7%, respectively). Preferred game genres were different; IGD subjects preferred simulation/strategy (28.5%) and RPG (27.3%) genres while non-IGD subjects did puzzle, arcade, and board games (31.0%). The main motives for gaming were different as well; the proportions of gamers who played for fun and for killing time were higher in non-IGD subjects (43.5% vs. 37.4%, 25.2% vs. 16.4%, respectively) while the proportion of gamers who played for relieving stress and for the sense of achievement was higher in IGD subjects (25.5% vs. 20.1%, and 15.2% vs. 8.2%, respectively). Scores of YIAT, SAS-SV, and BSCS and the prevalence of depression, GAD, AUD, and nicotine dependence were significantly higher among IGD subjects. 

We performed two additional subgroup analyses to explore the gender role in IGD. First, we compared between IGD and non-IGD subjects for males and females separately (see Table S1 in the Supplementary Materials), and then between males and females in the IGD group (see Table S2 in the Supplementary Materials). The difference of education levels and occupational status between IGD and non-IGD groups was not observed in females, whereas that of preferred game genre was not found in males. Other findings were comparable to the findings of the whole sample. When compared between the male IGD group and female IGD group, the male IGD group had a higher proportion of individuals who currently had full-time jobs, game community membership, and nicotine dependence, and spent more money on gaming, while the female IGD group had higher scores for SAS-SV and BSCS, indicating lower self-control ability, and higher prevalence of AUD than the male IGD group. Gaming device usage patterns differed between genders. PC > SM (448, 28.9%) and PC only (431, 27.8%) patterns were more prevalent in males, and these patterns persisted in the male IGD group. Females had a higher proportion of PC < SM (417, 27.6%) and SM only (505, 33.4%) patterns, while the female IGD group showed higher PC < SM and PC = SM patterns.

### 3.2. Comparison across Different Gaming Device Usage Patterns 

Table 2 shows the results of a comparison analysis across the five different gaming device usage groups. The mean age was highest in the SM only group and males were most prevalent in the PC only and PC > SM groups. PC only and PC = SM groups had higher prevalence of individuals with lower education. The PC only and PC > SM groups had higher proportions of students whereas the SM only and PC = SM groups had higher proportions of individuals who currently had full-time jobs. IGD was most prevalent in the PC = SM group (21.8%), followed by the PC > SM (15.5%) and PC < SM (13.9%) groups, and least prevalent in the SM only (6.6%) group. Time and money spent on gaming was highest in the PC > SM group and lowest in the SM only group. The PC > SM and PC = SM groups had the highest prevalence of game community membership and experience of offline meeting attendance. Preferred game genres and motives for gaming were different across groups. The SM only group preferred puzzle, arcade, and board games while the other groups preferred simulation/strategy and RPG games. PC predominant groups (PC only, PC > SM, PC = SM groups) mainly played games “for fun” and “for relieving stress” while SM predominant groups (PC < SM and SM only groups) chose to play games more “for killing time”. The SM only group had the lowest scores on YIAT, SAS-SV, and BSCS and proportions of depression, GAD, AUD, and nicotine dependence and PC = SM had the highest scores and proportions of comorbid psychopathology.

### 3.3. Predictive Value of the Gaming Device Usage Patterns

Table 3 shows the results of a binary logistic regression analysis predicting IGD using age, gender, time and money spent on gaming game community membership, device usage patterns, the scores of SAS-SV and BSCS, and the presence of depression, GAD, AUD, and nicotine dependence as covariates. Since the ranges of time and money spent on gaming were too broad to calculate odds ratios, these variables were divided dichotomously according to the median value: time spent on gaming during weekday (0: Minimum to 90 min, 1: 91 min to maximum) and weekend (0: Minimum to 150 min, 1: 151 min to maximum), and money (0: Minimum to KRW2000 (approximately 1.84 USD (1 USD = KRW1088.00, December 2017), 1: KRW2001 to maximum). When analyzed in a single equation, the input variables accounted for 39.5% of the total variance (Nagelkerke *R*^2^ = 0.395) and the prediction success was 89.1%. 

Individuals with higher age (B = 0.032, OR = 1.033), who spent 150 min or more on gaming during the weekend (B = 0.542, OR = 1.720), who spent more than KRW2001 on gaming per month (B = 0.729, OR = 2.072) and who owned a game community membership (B = 0.851, OR = 2.341) were likely to become IGD. As for the gaming device usage patterns, the PC = SM pattern increased the probability of IGD in comparison to the reference (PC only) group (B = 0.457, OR = 1.579). Individuals with higher SAS-SV and BSCS scores and depression were more prone to become IGD (B = 0.090, 0.043, and 0.667, OR = 1.094, 1.044, and 1.949 for SAS-SV, BSCS, and depression, respectively). 

## 4. Discussion

### 4.1. Characteristics of IGD in Smartphone Era

Overall, the prevalence of IGD was 12.9% in this study. The prevalence of IGD ranged from 0.6% to 46% depending on the sample and methods [29]. According to a Korean national survey conducted in 2015, the prevalence of Internet addiction was 5.8% in an adult population with ages ranging from 20 to 59 years old [7], which was lower than our results. Given that our sample was recruited from online survey responders who played online games and included those age ranged from 20 to 39, it seems reasonable that the prevalence of IGD is higher in our sample, though a direct comparison is impossible due to the differences in the sample collecting methods and applied diagnostic criteria. Rather, this result was in line with a previous study reporting that 38.7% of adult MMORPG players were IGD [30], since 20.5% of the responders played RPGs, most of which were MMORPGs. The prevalence of IGD is higher in Korea than in the other countries. The prevalence of IGD has been considered to be high in East Asian countries [31]. Cultural differences, such as Internet accessibility (speed of Internet access, availability of Wi-Fi, the cost paid for using Internet, or accessibility of Internet cafes), social norms for Internet gaming and device usage patterns, and government regulations for Internet gaming, as well as the sample recruitment and applied measurements may account for the different prevalence across different countries. Considering that IGD subjects in this study showed comparable clinical characteristics to previous studies, such that they owned game community memberships and attended offline meetings more frequently, spent more time and money on gaming, showed more preference for the simulation/strategy and RPG genres, were motivated to play games more to relieve stress, manifested higher degrees of smartphone addiction and lower levels of self-control, and had higher prevalence of depression, GAD, AUD, and nicotine dependence than non-IGD individuals [3,6,32,33,34,35,36], our sample may represent the general IGD population. In contrast to prior findings that IGD was frequently observed in male adolescents [37,38], the proportion of females in the IGD group was 44.4% and the mean age was higher among those with IGD. Given that our sample only included an adult population, this would be a unique feature of adult IGD, distinct from adolescent IGD. The spread of smartphone games, which provide easier accessibility and portability than PC games, may increase the number of female and middle-aged adult gamers. 

Some gaming-related and clinical characteristics were significantly different between male and female participants. Preferred game genre and reason for gaming were different between genders, in line with previous findings [39,40,41]. Males displaying IGD spent more money on gaming and had more game community memberships, both classical features of IGD, while females displaying IGD had higher smartphone addiction severity and lower self-control ability, which may indicate the role of problematic smartphone use on IGD in females [42,43]. The proportion of nicotine dependence was higher among males with IGD, while that of AUD was higher among females with IGD. This may be due to an increased risk of AUD in the presence of comorbid psychiatric condition in females [44]. Gaming device usage patterns were significantly different between genders as well; males had a tendency to play PC games more than SM games while females seemed to play more SM games. The features of each device, which are described below, differences in time spent on gaming, preference of specific game genres that were more suitable for PC interface, and social expectation toward gaming behavior between genders may account for the results [41].

### 4.2. Characteristics across Different Gaming Device Usage Patterns 

When comparing across different gaming device usage patterns, several intriguing findings were observed. First, IGD was more prevalent in combined user groups (PC > SM, PC = SM, and PC < SM groups), especially in the PC = SM group, than the single user groups (PC and SM only). IGD is a behavioral addiction that psychologically and neurobiologically resembles substance addiction [3]. As substance abusers frequently co-administer multiple substances simultaneously [45], IGD subjects may need multiple methods of playing games. In particular, the PC = SM group had the highest prevalence of IGD, possibly due to the differences in motivational background and gaming interface of each device. Though both PC and SM games provide a sense of reward and relatedness as well as escape from negative emotions [46,47], each device may satisfy different needs. Since PC games usually provide high quality sound and visual effects and necessitate substantial duration for playing, they may have the potential to provide a sense of immersion, achievement, and competitiveness [47]. Meanwhile, SM games may increase a sense of social relatedness and attenuate loneliness and negative emotion [48], since SM games are easily played in association with social networking service (SNS) applications. In this study, motives for gaming were significantly different across groups; PC predominant gamers played games “for fun”, “for relieving stress”, and “for achievement”, while SM predominant gamers played games “for killing time”, “for fun”, and “for relieving stress”. Considering that the PC = SM group manifested more comorbid psychopathology, which can be taken as being more defective in self-soothing ability, they may need both devices to satisfy different needs and become more indulged in gaming than the other groups. 

Second, each combined user group (PC > SM, PC = SM, and PC < SM groups) had unique clinical characteristics that may modify the course and prognosis of IGD. Individuals in the PC > SM group showed typical behavioral manifestations of IGD [32]. They were younger, had a higher male proportion, spent more time and money on gaming, and had more game community memberships, but had less prevalence of comorbid psychopathology than other combined user groups. Meanwhile, the PC = SM group showed the highest prevalence of comorbid psychopathology, implying that they may be suffering from more difficulty in academic or occupational adaptation and impelled to continuously play games to soothe their negative emotions or to alleviate substance craving. The PC < SM group manifested similar gaming behaviors to the SM only group while comorbidity patterns were comparable to the PC = SM group. These findings suggest an important clinical implication that detailed investigations of the gaming device usage patterns may provide precise estimation of current status and prediction of prognosis. 

Third, the PC only group spent less time and money on gaming and showed lower scores on YIAT and SAS-SV than the combined user groups. PC games usually need special environmental requisites, such as fast online connection speeds, high-resolution large screens, and charged memberships, which may paradoxically keep the PC gamers away from playing anytime and anywhere. In addition, since PC games offer excellent visual and sound effects and sophisticated game platforms, PC gamers may not be satisfied with SM games. Another interesting finding was that PC only gamers manifested as high prevalence of depression, GAD, and AUD as the PC < SM group. Considering that PC games had more addictive potential than SM games [49,50], PC only gamers may have difficulty in functioning as is the case for the combined users despite less behavioral disturbances.

Fourth, the SM only group had a higher mean age and proportion of females, spent less time and money on gaming, and had lower severity of Internet and smartphone addiction and prevalence of IGD, depression, GAD, AUD, and nicotine dependence and higher self-control ability than the other groups. Explanations can be drawn from the unique characteristics of SM game interfaces and smartphones themselves. First is the structural characteristic of smartphone games. Despite substantial technical advances that enable sophisticated games to be played on smartphone platforms, simpler games such as puzzle, arcade, or board games are still more suitable and preferred for the SM gamers, possibly due to unchangeable structures such as small screens and viewing angles, which interfere with the sense of immersion [51]. Secondly, the emergence of the social networking games (SNGs), a hybrid game genre that connects games with SNSs, should be considered. SNG players can play games and interact with online friends simultaneously within existing SNS applications. Many responders in the SM only group answered that they preferred to play SNGs, such as “Farmville” or “I Love Coffee”, to mention but a few. Considering that getting something useful out of playing, such as the improvement of relationships, was the major motive to play SNGs [52], SM gamers, especially SNG players, may prefer to play games to get something that classical PC games cannot provide, such as enhancement of social relatedness and the alleviation of feelings of loneliness. Third, the unique properties of smartphones themselves can be considered. In addition to the portability and availability for easy and frequent access that leads to habitual checking behavior [53], the multi-tasking function enables SM users to play games while they are doing other things, such as searching on websites or sending messages. Though SM gamers may not spend as much time and money on gaming and feel as much immersion in games as PC gamers do, they may use games to “kill time” between tasks or during “empty” hours of waiting. 

### 4.3. The Role of Gaming Device Usage Patterns on IGD

Logistic regression analysis revealed that the PC = SM pattern was a predictor of IGD, along with time and money spent on gaming, game community membership, the severity of smartphone addiction, self-control ability, and the presence of depression. As mentioned above, individuals in the PC = SM group had the highest prevalence of comorbidity and thus they may feel that it is difficult to quit games probably due to lack of tolerability for withdrawal symptoms or negative emotions. Another possibility lays in that the PC = SM pattern may have an intrinsic risk potential for IGD since no multicollinearity was observed among variables. Further studies are necessary to elucidate the neurobiology that underlies this distinct device usage pattern. An important finding that higher financial investment was associated with increased risk of IGD should be marked. This was in line with previous findings demonstrating that higher money spent on gaming was associated with IGD in adolescents and had a predictive value of IGD in adults [32,54]. Most online games are freemium services, which are free for download, but require payment for additional features or virtual goods [55]. Even though the amount is small, as much as $2, it could cause a big financial problem if accumulated, as is the case of gambling disorder, and could induce more commitment toward the games. Increased risk of IGD with individuals who spent more than 2000 won (approximately $1.84) for gaming per month would support the danger of accumulated micropayments.

There are some limitations that should be noted. First, the causality of IGD and the device usage patterns were not elucidated due to the cross-sectional nature of this study. Second, the distinction between the game genres may not be mutually exclusive because of the fast evolution of hybrid game genres. For example, Cookie Run is a hybrid form of running and action games. Likewise, the distinction between the games and other smartphone applications were not exclusive, such as with SNGs. Further research studies need to investigate the role of hybrid games or applications on IGD or other technological addiction. Third, IGD was determined by self-reports of endorsement to five or more DSM-5 IGD criteria, which was originally developed for professional usage. However, Lemmens and colleagues have demonstrated solid psychometric property and high practicality of The Short, Nine-Item IGD Scale, which assessed IGD using the self-reporting of nine items of DSM-5 IGD criteria with a dichotomous scale [12]. Despite deviation from original professional purposes, the self-rating IGD would be a valid tool for identifying IGD, especially in a large sample. Fourth, since comorbid psychopathology and gaming device usage patterns were assessed via self-report, over- or under-estimation of psychopathology and a recall bias may be present. Overestimation of IGD should be considered when determining IGD by the self-report as well. As mentioned above, the prevalence of IGD was higher in this study than in the national survey. Further research studies may need to validate the findings using diagnostic interviews and machinery collecting methods. Lastly, we did not include console users. In contrast to the United States of America and European countries, only a small number of gamers used video and portable consoles for gaming [1]. Console games had different features than PC and SM games, such as the need for buying consoles and software, and may manifest unique gaming-relate behaviors and characteristics. Further studies are necessary to investigate the characteristics of console gamers, especially in comparison with PC and SM gamers. Alternatively, was this meant to be “the United States of America”? If so, please specify to avoid potential ambiguity with the continents.

## 5. Conclusions

In this study, we investigated how gaming device usage patterns influenced gaming behaviors and clinical characteristics. To the best of our knowledge, this is the first study to elucidate the differences in gaming behavior and comorbid psychopathology across gaming device usage patterns and the role of specific usage patterns on IGD. Despite limitations, this study is notable for having been conducted using a large sample and having covered a diverse range of gaming behaviors and clinical characteristics, and thus contributes to deepening our understanding of IGD in the smartphone era. Our findings suggest that gaming device usage patterns may be associated with the occurrence, course, and prognosis of IGD. An important clinical implication can be drawn that the evaluation of gaming device usage patterns would help to determine the risk, predict outcome, and offer optimized treatment options for IGD.

## Figures and Tables

**Table 1:** Comparison between the IGD and non-IGD groups.

Variables	IGD	Non-IGD	X^2^/U	*p*
N (%)	396 (12.9%)	2662 (87.1%)		
Age	27.63 ± 5.797	26.85 ± 5.862	572,613.000	0.005 *
Male (%)	220 (55.6%)	1328 (49.9%)	4.431	0.035 *
Education levels				
Up to 12 years (high school)	63 (15.9%)	276 (10.4%)	10.731	0.001 **
More than 13 years	333 (84.1%)	2386 (89.6%)		
Occupational status				
Student	123 (31.1%)	1029 (38.7%)	8.472	0.014 *
Current full-time job	213 (53.8%)	1227 (48.0%)		
No currently full-time job	60 (15.2%)	356 (13.4%)		
Time spent on gaming				
Weekday (min)	167.79 ± 124.190	107.63 ± 96.227	722,949.000	0.000 **
Weekend (min)	253.76 ± 152.207	169.02 ± 131.868	729,658.000	0.000 **
Money spent on gaming (KRW)	32,270.45 ± 48,491.203	11,599.61 ± 27,190.750	752,279.500	0.000 **
Game community membership	253 (63.9%)	818 (30.7%)	166.566	0.000 **
Ever attended offline meeting	179 (45.2%)	352 (13.2%)	59.393	0.000 **
Gaming device usage pattern				
PC only	87 (22.0%)	633 (23.8%)	51.909	0.000 **
PC > SM	90 (22.7%)	490 (18.4%)		
PC = SM	71 (17.9%)	255 (9.6%)		
PC < SM	102 (25.8%)	633 (23.8%)		
SM only	46 (11.6%)	651 (24.5%)		
Preferred game genre				
Simulation/strategy	113 (28.5%)	708 (26.6%)	23.102	0.000 **
RPG	108 (27.3%)	521 (19.6%)		
Sports/racing	60 (15.2%)	413 (15.5%)		
Shooting/action	33 (8.3%)	196 (7.4%)		
Puzzle/arcade/board game	82 (20.7%)	824 (31.0%)		
Reason for gaming				
For fun	148 (37.4%)	1159 (43.5%)	44.352	0.000 **
For killing time	65 (16.4%)	671 (25.2%)		
For relieving stress	101 (25.5%)	535 (20.1%)		
For need	22 (5.6%)	80 (3.0%)		
For achievement	60 (15.2%)	217 (8.2%)		
YIAT	53.98 ± 26.267	36.80 ± 18.800	788,470.500	0.000 **
SAS-SV	39.97 ± 9.015	28.75 ± 10.114	838,582.000	0.000 **
BSCS	53.98 ± 26.267	36.80 ± 18.800	775,457.500	0.000 **
Depression (%)	252 (63.6%)	631 (23.7%)	267.652	0.000 **
Generalized anxiety disorder (%)	179 (45.2%)	383 (14.4%)	218.205	0.000 **
Alcohol use disorder (%)	139 (35.1%)	446 (16.8%)	75.003	0.000 **
Nicotine dependence (%)	55 (59.8%)	133 (31.7%)	25.675	0.000 **

Abbreviations: IGD: Internet Gaming Disorder; KRW: Korean Won; PC: Personal computer; SM: Smartphone; RPG: Role-playing game; YIAT: Young’s Internet Addiction Test; SAS-SV: Smartphone Addiction Scale-Short Form; BSCS: Brief Self-Control Scale. * *p* < 0.05, ** *p* < 0.005.

**Table 2:** Differences across device usage patterns.

Variables	PC Only	PC > SM	PC = SM	PC < SM	SM Only	X^2^/H	*p*	Post-Hoc
N (%)	720 (23.5%)	580 (19.0%)	326 (10.7%)	735 (24.0%)	697 (22.8%)			
Age	25.70 ± 5.306	25.74 ± 5.458	27.16 ± 5.800	27.11 ± 5.890	28.95 ± 6.142	140.747	0.000 **	e > a,b,c,d, a,b < c,d
Male (%)	448 (62.2%)	431 (74.3%)	159 (48.8%)	318 (43.3%)	192 (27.5%)	333.801	0.000 **	
Education levels								
Up to 12 years	95 (13.2%)	66 (11.4%)	46 (14.1%)	65 (8.8%)	67 (9.6%)	11.608	0.021 *	
More than 13 years	625 (86.8%)	514 (88.6%)	280 (85.9%)	670 (91.2%)	630 (90.4%)			
Occupational status								
Student	330 (45.8%)	292 (50.3%)	107 (32.8%)	254 (34.6%)	169 (24.2%)	134.837	0.000 **	
Current full-time job	281 (39.0%)	217 (37.4%)	180 (55.2%)	383 (42.1%)	429 (61.5%)			
No current full-time job	109 (15.1%)	71 (12.2%)	39 (12.0%)	98 (13.3%)	99 (14.2%)			
IGD (%)	87 (12.1%)	90 (15.5%)	71 (21.8%)	102 (13.9%)	46 (6.6%)	51.909	0.000 **	
Time spent on gaming								
Weekday (min)	111.65 ± 103.703	139.56 ± 105.489	120.45 ± 112.919	110.11 ± 87.180	96.75 ± 103.262	122.254	0.000 **	e < a,b,c,d, b > a,c,d
Weekend (min)	194.60 ± 148.341	220.82 ± 134.656	180.53 ± 124.000	172.78 ± 123.798	137.61 ± 135.296	226.227	0.000 **	e < a,b,c,d, b > a,c,d
Money spent on gaming (KRW)	17,298.34 ± 37,995.779	24,993.36 ± 36,335.539	19,647.24 ± 34,119.854	11,973.13 ± 29,297.559	2082.50 ± 7421.816	577.364	0.000 **	e < a,b,c,d, b > a,c,d d < a,c
Game community membership	220 (30.6%)	304 (52.4%)	153 (46.9%)	273 (37.7%)	117 (16.8%)	207.871	0.000 **	
Ever attended offline meeting	101 (45.9%)	172 (56.6%)	88 (57.5%)	128 (46.2%)	42 (35.9%)	21.019	0.000 **	
Preferred game genre								
Simulation/strategy	255 (35.4%)	230 (39.7%)	77 (23.6%)	152 (20.7%)	107 (15.4%)	591.480	0.000 **	
RPG	183 (25.4%)	132 (22.8%)	79 (24.2%)	157 (21.4%)	78 (11.2%)			
Sports/racing	119 (16.5%)	89 (15.3%)	69 (21.2%)	123 (16.7%)	73 (10.5%)			
Shooting/action	88 (12.2%)	59 (10.2%)	21 (6.4%)	40 (5.4%)	12 (3.0%)			
Puzzle/arcade/board game	75 (10.4%)	70 (12.1%)	80 (24.5%)	263 (29.0%)	418 (60.0%)			
Reason for gaming								
For fun	237 (45.4%)	267 (46.0%)	147 (45.1%)	318 (43.3%)	248 (35.6%)	164.244	0.000 **	
For killing time	120 (16.7%)	88 (15.2%)	66 (20.2%)	181 (24.6%)	281 (38.2%)			
For relieving stress	178 (24.7%)	134 (23.1%)	69 (21.2%)	153 (20.8%)	102 (14.6%)			
For need	34 (4.7%)	24 (4.1%)	13 (4.0%)	21 (2.9%)	10 (1.4%)			
For achievement	61 (8.5%)	67 (11.6%)	31 (9.5%)	62 (8.4%)	56 (8.0%)			
YIAT	38.65 ± 20.899	43.56 ± 18.530	44.08 ± 21.511	40.73 ± 20.591	31.36 ± 19.930	145.760	0.000 **	e < a,b,c,d, a < b,c
SAS-SV	29.50 ± 10.646	30.19 ± 11.029	31.58 ± 11.149	31.60 ± 10.392	28.76 ± 10.657	31.614	0.000 **	e < c,d, a < c,d
BSCS	36.51 ± 6.663	36.17 ± 6.959	36.73 ± 6.920	36.49 ± 6.742	35.33 ± 6.795	17.479	0.002 **	e < a,c,d
Depression (%)	218 (30.3%)	156 (26.9%)	116 (35.6%)	233 (31.7%)	160 (23.0%)	23.687	0.000 **	
Generalized anxiety disorder (%)	141 (19.6%)	101 (17.4%)	74 (22.7%)	144 (19.6%)	102 (14.6%)	12.350	0.015 *	
Alcohol use disorder (%)	141 (31.6%)	84 (22.0%)	82 (36.4%)	160 (31.3%)	118 (26.3%)	19.325	0.001 **	
Nicotine dependence (%)	35 (31.0%)	50 (38.8%)	23 (44.2%)	60 (43.2%)	20 (25.3%)	10.006	0.040 *	

Abbreviations: PC: Personal computer; SM: Smartphone; IGD: Internet Gaming Disorder; KRW: Korean Won; RPG: Role-playing game; YIAT: Young’s Internet Addiction Test; SAS-SV: Smartphone Addiction Scale Short Form; BSCS: Brief Self-Control Scale. * *p* < 0.05, ** *p* < 0.005.

**Table 3:** Logistic regression results predicting Internet gaming disorder.

Variables	B (s.e.)	OR	95% CI	*p*
Age	0.032 (0.011)	1.033	1.010–1.056	0.005 *
Gender (male)	0.260 (0.144)	1.296	0.977–1.719	0.072
Weekday gaming hour (>90 min)	0.262 (0.144)	1.300	0.931–1.816	0.124
Weekend gaming hour (>150 min)	0.542 (0.170)	1.720	1.232–2.399	0.001 **
Money spent on gaming (>KRW2000)	0.729 (0.144)	2.072	1.562–2.749	0.000 **
Game community membership	0.851 (0.137)	2.341	1.791–3.061	0.000 **
Gaming device usage pattern				
PC only	Reference			0.000 **
PC > SM	−0.085 (0.196)	0.919	0.626–1.349	0.666
PC = SM	0.457 (0.213)	1.579	1.040–2.397	0.032 *
PC < SM	0.019 (0.189)	1.019	0.703–1.476	0.921
SM only	−0.144 (0.229)	0.866	0.552–1.357	0.529
SAS-SV	0.090 (0.008)	1.094	1.076–1.112	0.000 **
BSCS	0.043 (0.013)	1.044	1.018–1.070	0.001 **
Depression	0.667 (0.168)	1.949	1.403–2.708	0.000 **
Generalized anxiety disorder	0.132 (0.173)	1.141	0.812–1.603	0.447
Alcohol use disorder	0.254 (0.150)	1.2899	0.961–1.728	0.090
Nicotine dependence	0.384 (0.223)	1.468	0.949–2.271	0.085

Abbreviations: s.e.: standard error; KRW: Korean Won; PC: Personal computer; SM: Smartphone; SAS-SV: Smartphone Addiction Scale Short Form; BSCS: Brief Self-Control Scale; OR: odds ratio; CI: confidence interval. * *p* < 0.05, ** *p* < 0.005.

## Supplementary Materials

The following are available online at www.mdpi.com/1660-4601/14/12/1512/s1, Table S1. Comparison between the IGD and non-IGD groups in each gender group; Table S2. Comparison between males and females in the IGD group.
