# Supplementary material for: Gaming Device Usage Patterns Predict Internet Gaming Disorder: Comparison across Different Gaming Device Usage Patterns
Source: Int J Environ Res Public Health. 2017 Dec 5;14(12):1512. doi: 10.3390/ijerph14121512 (PMC5750930; doi:10.3390/ijerph14121512)
Supplement: Supplementary file 1 [file ijerph-14-01512-s001.pdf]

Table S1. Comparison between the IGD and non-IGD groups in each gender group.

Variables	Male (n = 1548)				Female (n = 1510)			
	IGD	Non-IGD	X^s/U	*p*	IGD	Non-IGD	X^2/U	*p*
**N (%)**	220 (14.2%)	1328 (85.8%)			176 (11.7%)	1334 (88.3%)		
Age	28.25 ± 6.191	27.51 ± 6.363	135656.000	0.088	26.87 ± 5.180	26.19 ± 5.237	106794.500	0.050
Education levels								
Up to 12 years (high school)	41 (18.6%)	139 (10.5%)	12.258	0.000**	22 (12.5%)	137 (10.3%)	0.821	0.365
More than 13 years	179 (81.4%)	1189 (89.5%)			154 (87.5%)	1197 (89.7%)		
Occupational status								
Student	70 (31.8%)	586 (44.1%)	11.710	0.003**	53 (30.1%)	443 (33.2%)	1.046	0.593
Current full-time job	126 (57.3%)	624 (47.0%)			87 (49.4%)	653 (49.0%)		
No current full-time job	24 (10.9%)	118 (8.9%)			36 (20.5%)	238 (17.8%)		
Time spent on gaming								
Weekday (min)	175.24 ± 127.200	115.18 ± 98.881	93502.000	0.000**	158.49 ± 120.033	100.12 ± 92.940	7331.000	0.000**
Weekend (min)	262.12 ± 158.218	175.24 ± 127.200	95548.500	0.000**	243.31 ± 144.106	154.92 ± 126.231	68113.500	0.000**
Money spent on gaming (KRW)	38646.82 ± 52282.118	16774.89 ± 33467.926	90360.000	0.000**	24300.00 ± 42092.216	6447.60 ± 17538.361	61793.000	0.000**
Game community membership	152 (69.1%)	505 (38.0%)	74.552	0.000**	101 (57.4%)	313 (23.5%)	89.914	0.000**
Ever attended offline meeting	111 (50.5%)	238 (17.9%)	31.466	0.000**	68 (38.6%)	114 (8.5%)	29.605	0.000**
Gaming device usage pattern								
PC only	54 (24.5%)	394 (29.7%)	14.199	0.007*	33 (18.8%)	239 (17.9%)	37.316	0.000**
PC > SM	68 (30.9%)	363 (27.3%)			22 (12.5%)	127 (9.5%)		
PC = SM	35 (15.9%)	124 (9.3%)			36 (20.5%)	131 (9.8%)		
PC < SM	45 (20.5%)	273 (20.6%)			57 (32.4%)	360 (27.0%)		
SM only	18 (8.2%)	174 (13.1%)			28 (15.9%)	477 (35.8%)		
Preferred game genre								
Simulation/strategy	82 (37.3%)	505 (38.0%)	7.523	0.111	31 (17.6%)	203 (15.2%)	14.384	0.006*
RPG	62 (28.2%)	274 (20.6%)			46 (26.1%)	247 (18.5%)		
Sports/racing	33 (15.0%)	232 (17.5%)			27 (15.3%)	181 (13.6%)		
Shooting/action	19 (8.6%)	124 (9.3%)			14 (8.0%)	72 (5.4%)		
Puzzle/arcade/board game	24 (10.9%)	193 (14.5%)			58 (33.0%)	631 (47.3%)		
Reason for gaming								
For fun	81 (36.8%)	579 (43.6%)	28.245	0.000**	67 (38.1%)	580 (43.5%)	17.052	0.002**
For killing time	27 (12.3%)	279 (21.0%)			38 (21.6%)	392 (29.4%)		
For relieving stress	57 (25.9%)	290 (21.8%)			44 (25.0%)	245 (18.4%)		
For need	15 (6.8%)	61 (4.6%)			7 (4.0%)	19 (1.4%)		
For achievement	40 (18.2%)	119 (9.0%)			20 (11.4%)	98 (7.3%)		
YIAT	53.90 ± 25.948	38.43 ± 18.137	77560.000	0.000**	54.09 ± 26.734	35.18 ± 19.308	55948.000	0.000**
SAS-SV	38.93 ± 9.404	27.28 ± 10.174	58513.000	0.000*	41.27 ± 8.348	30.21 ± 9.843	46212.500	0.000**

BSCS	40.16 ± 6.278	34.90 ± 6.481	80283.500	0.000**	42.14 ± 5.798	36.04 ± 6.695	57190.000	0.000**
Depression (%)	134 (60.9%)	278 (18.0%)	154.419	0.000**	118 (67.0%)	353 (26.5%)	119.320	0.000**
Generalized anxiety disorder (%)	99 (45.0%)	178 (13.4%)	128.245	0.000**	80 (45.5%)	205 (15.4%)	91.924	0.000**
Alcohol use disorder (%)	58 (26.4%)	121 (9.1%)	54.932	0.000**	81 (46.0%)	325 (24.4%)	37.108	0.000**
Nicotine dependence (%)	44 (20.0%)	106 (8.0%)	31.150	0.000**	11 (6.3%)	27 (2.0%)	11.319	0.001**

Abbreviations: IGD: Internet Gaming Disorder; KRW: Korean Won; PC: Personal computer; SM: Smartphone; RPG: role-playing game; YIAT: Young's Internet Addiction Test; SAS-SV: Smartphone Addiction Scale-Short Form; BSCS: Brief Self-Control Scale. * $p < 0.05$, ** $p < 0.005$

Table S2. Comparison between males and females in the IGD group.

**Variables**	Male	Female	X^2/U	*p*
N (%)	220 (14.2%)	176 (11.7%)		
Age	28.25 ± 6.191	26.87 ± 5.180	17362.000	0.077
Education levels				
Up to 12 years (high school)	41 (18.6%)	22 (12.5%)	2.752	0.097
More than 13 years	179 (81.4%)	154 (87.5%)		
Occupational status				
Student	70 (31.8%)	53 (30.1%)	7.089	0.029*
Current full-time job	126 (57.3%)	87 (49.4%)		
No current full-time job	24 (10.9%)	36 (20.5%)		
Time spent on gaming				
Weekday (min)	175.24 ± 127.200	158.49 ± 120.033	17761.500	0.155
Weekend (min)	262.12 ± 158.218	243.31 ± 144.106	18297.000	0.346
Money spent on gaming (KRW)	38646.82 ± 52282.118	24300.00 ± 42092.216	15035.000	0.000**
Game community membership	152 (69.1%)	101 (57.4%)	5.806	0.016*
Ever attended offline meeting	111 (50.5%)	68 (38.6%)	.953	0.329
Gaming device usage pattern				
PC only	54 (24.5%)	33 (18.8%)	27.632	0.000**
PC > SM	68 (30.9%)	22 (12.5%)		
PC = SM	35 (15.9%)	36 (20.5%)		
PC < SM	45 (20.5%)	57 (32.4%)		
SM only	18 (8.2%)	28 (15.9%)		
Preferred game genre				

Simulation/strategy	82 (37.3%)	31 (17.6%)	36.404	0.000**
RPG	62 (28.2%)	46 (26.1%)		
Sports/racing	33 (15.0%)	27 (15.3%)		
Shooting/action	19 (8.6%)	14 (8.0%)		
Puzzle/arcade/board game	24 (10.9%)	58 (33.0%)		
Reason for gaming				
For fun	81 (36.8%)	67 (38.1%)	9.665	0.046*
For killing time	27 (12.3%)	38 (21.6%)		
For relieving stress	57 (25.9%)	44 (25.0%)		
For need	15 (6.8%)	7 (4.0%)		
For achievement	40 (18.2%)	20 (11.4%)		
YIAT	53.90 ± 25.948	54.09 ± 26.734	19349.000	0.992
SAS-SV	38.93 ± 9.404	41.27 ± 8.348	16412.000	0.009*
BSCS	40.16 ± 6.278	42.14 ± 5.798	15759.500	0.001**
Depression (%)	134 (60.9%)	118 (67.0%)	1.591	0.207
Generalized anxiety disorder (%)	99 (45.0%)	80 (45.5%)	.008	0.928
Alcohol use disorder (%)	58 (26.4%)	81 (46.0%)	16.589	0.000**
Nicotine dependence (%)	44 (20.0%)	11 (6.3%)	15.457	0.000**

Abbreviations: IGD: Internet Gaming Disorder; KRW: Korean Won; PC: Personal computer; SM: Smartphone; RPG: role-playing game; YIAT: Young's Internet Addiction Test; SAS-SV: Smartphone Addiction Scale-Short Form; BSCS: Brief Self-Control Scale. * $p < 0.05$, ** $p < 0.005$.
